# AI-driven troubleshooting for TrueBeam systems: Development and testing of a GPT-4.1 Chatbot

**DOI:** 10.1016/j.tipsro.2026.100420

**Published:** 2026-07-15

**Authors:** Cory Knill, Sean Devan, Charles Matrosic, Jill Moreau, Zheng Zhang

**Affiliations:** Department of Radiation Oncology, University of Michigan, Ann Arbor, MI, United States of America

**Keywords:** LINACs, Troubleshooting, AI, Chatbots

## Abstract

**Purpose:**

Troubleshooting linear accelerator faults during patient care is time-critical and cognitively demanding. Access to relevant historical information is often slow and experience-dependent. To streamline information retrieval and support decision-making, a TrueBeam troubleshooting chatbot powered by a large language model (LLM) was developed and tested.

**Methods:**

Troubleshooting records from five TrueBeam linacs over eight years were extracted from an in-house database. After removing non-UTF-8 characters and normalizing formatting, each issue was stored as a structured text file for retrieval. Files were indexed in a GPT-4.1–based environment, with parameters (e.g., temperature, retrieved chunks) iteratively tuned. Performance was evaluated using standardized questions across domains including recall, real-time troubleshooting, aggregation, safety, and temporal filtering. Four physicists scored responses using a predefined rubric.

**Results:**

A total of 1394 logs (5.4 MB) were indexed, with indexing completed in 16 min. Mean response time was 7.5 ± 2.5 s. The chatbot performed well in retrieving prior events, summarizing institutional experience, and recognizing when information was unavailable. Performance was largely insensitive to temperature and chunk number, except under severely limited retrieval. Weaknesses included occasional procedural misordering, unclear responsibility between physicists and service personnel, verbosity, and inconsistent temporal filtering.

**Conclusion:**

A GPT-4.1–based RAG chatbot can rapidly surface relevant institutional knowledge for linac troubleshooting and may reduce cognitive burden during machine faults. However, important safety and workflow risks remain. Such systems should function as decision-support tools and require explicit guardrails, role definition, and formal risk evaluation prior to broad clinical deployment.

## Highlights

• AI chatbot developed for real-time linac fault troubleshooting.
• Reformats historical machine logs to enhance retrieval accuracy.
• Delivers context-specific guidance from prior machine faults.
• Assists physicists with rapid, data-driven troubleshooting.
• Enhances efficiency and consistency in clinical machine support.

## Introduction

Unplanned linear accelerator (linac) faults are a persistent challenge in radiation oncology and represent critical clinical events with direct operational and patient-care consequences. [1], [2], [3] When a linac goes offline, even brief outages can lead to treatment delays, cancellations, workflow disruption, and increased stress for patients and staff. [4], [5] In a field where treatment continuity is essential for optimal outcomes, rapid and accurate troubleshooting is therefore a key determinant of both care quality and operational efficiency. [6], [7], [8]

AAPM Task Group 314 provides structured guidance for managing linac faults, emphasizing defined roles, documentation, and safe recovery procedures. [9] Prior studies have shown that standardized fault reporting, rapid communication, and systematic use of machine data can significantly reduce response times, machine downtime, and treatment cancellations. [10] Despite these advances, troubleshooting in many centers remains largely manual and experience-dependent, relying on interpretation of cryptic error codes and time-consuming review of heterogeneous machine logs. This approach is difficult to scale, vulnerable to staff-turnover and knowledge loss, and often insufficient for addressing intermittent or unfamiliar faults. [11]

Recent work in industrial settings suggests that conversational artificial intelligence (AI) tools can improve diagnostic accuracy and reduce initial troubleshooting time, though challenges related to system integration and user acceptance remain. [12], [13] These findings suggest potential application in radiation oncology, where similar approaches may support clinical linac troubleshooting. [14] Linac machine logs, while currently underutilized due to their volume and heterogeneity, represent a rich repository of historical operational data and institutional knowledge that may be well suited to modern AI-based analysis. In parallel, AI tools have already been explored in radiation oncology for predicting QA failures, machine downtime, and optimizing machine schedules, among other applications, highlighting the broader role of AI in supporting clinical operations. [15], [16], [17] Within this context, a conversational tool could serve as a complementary component that integrates with these existing approaches as part of a broader clinical AI framework.

In this work, we present the development and evaluation of a machine troubleshooting chatbot for medical linear accelerators using a Retrieval-Augmented Generation (RAG) architecture. [18] This approach combines a large language model with a curated knowledge base derived from historical, semi-structured machine logs, allowing responses to be explicitly grounded in verifiable source data. The primary goal of the chatbot is to provide real-time troubleshooting assistance by identifying similar historical machine events and recommending actions that previously contributed to issue resolution. We describe the design, implementation, and evaluation of this RAG-based chatbot, highlighting both the potential benefits and the practical pitfalls of applying generative AI to support linac troubleshooting.

## Materials and methods

The design of the machine troubleshooting chatbot followed an iterative process, that included data collection, data formatting, model parameter selection and testing.

### Data collection

Indexing data consisted of historical troubleshooting logs authored by medical physicists and residents over an eight-year period. Records were drawn from five TrueBeam linacs across multiple hospitals and included both treatment unit and ancillary system issues. Logs were maintained within a legacy in-house database that predated installation of the earliest TrueBeam.

**Fig. 1:**
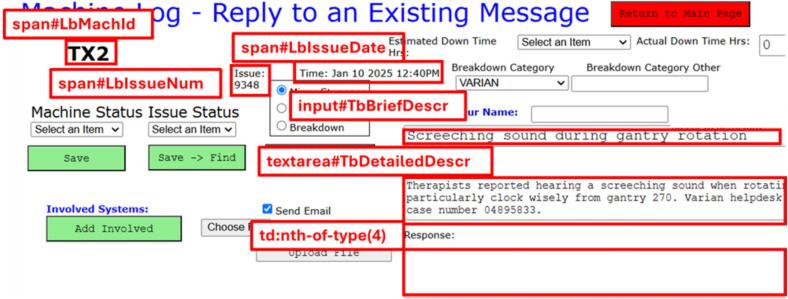
Example of a machine troubleshooting log entry accessed through a browser-based interface, with the fields used for model development highlighted in red. (For interpretation of the references to colour in this figure legend, the reader is referred to the web version of this article.)

Entries were accessed through a browser-based interface, with each issue documented on a single page using structured selections and free text. Several fields were inconsistently populated; therefore, only reliable elements were retained for model development: machine ID, issue ID, report date, brief title, initial description, and follow up comments (Fig. 1). A Python-based web scraper was developed to automate page navigation and data extraction. Google Chrome Developer Tools were used to identify page elements and guide code development.

### Data formatting & model indexing

The extraction process generated one spreadsheet per machine, with each issue represented as a row and selected data fields organized into columns. Files were converted to text for indexing within U-M Maizey, a University of Michigan software environment used to develop the GPT-4.1-based RAG model. Initial indexing was performed using default parameters.

Early indexing attempts failed because non-UTF-8 characters were incompatible with the model indexing. These characters, along with newline and tab markers, were removed through scripted preprocessing in Excel, after which the model trained successfully.

When training the RAG model, the machine logs are split into smaller chunks that are embedded and stored in a vector index to support similarity-based retrieval. Despite successful indexing, early queries revealed poor retrieval performance, with responses frequently merging unrelated events. This behavior was attributed to sequential chunking of the source material into fixed 1000-token segments without structural boundaries between logs. As a result, individual issues were not preserved during retrieval. Performance improved after iterative reformatting, in which each machine issue was stored as an independent structured text file, enabling clear separation of events and improved recall accuracy.

### Model parameter selection & testing

In addition to testing different text file formats, several model parameters were adjusted to identify the optimal configuration. Model optimization involved adjustment of the system prompt, temperature, and the number of retrieved chunks per response. Temperature controlled response variability, where 0 produced deterministic outputs and 1 yielded greater randomness. Increasing the number of chunks allowed retrieval from a larger set of source documents but increased response time. Preliminary testing using a selection of historical cases confirmed functionality and informed prompt refinement to improve answer quality.

Response time was evaluated using 10 standardized questions spanning four categories listed in Table 1. Questions were developed to represent a range of clinically relevant use cases, including troubleshooting scenarios that could arise during clinical operations as well as retrospective data-mining queries of historical logs. While retrospective queries were not the primary intended use of the model, they were included to evaluate performance for questions users may reasonably ask in practice. For chunk experiments, temperature was held at 0.4 while the number of chunks ranged from 1 to 100. For temperature experiments, the number of chunks was fixed at 40 while temperature varied from 0 to 1. Response times were recorded for all configurations, normalized to the maximum observed value for each question, and averaged across models. Standard deviations were calculated to quantify variability.

**Table 1:** Categories of test scenarios used to evaluate the Machine Troubleshooting Chatbot model and their corresponding purposes.

Category	Purpose
Historical Event Recall and Institutional Memory	Test whether the model can retrieve past occurrences and summarize prior actions rather than defaulting to generic guidance.
Real-Time Clinical / Operational Troubleshooting	Evaluate step-by-step procedural accuracy and whether responses stay within appropriate clinical scope.
Aggregation and Statistical Reasoning Over Documents	Stress-test the model's ability to synthesize across many records rather than retrieve a single document.
Safety, Policy, and “Out-of-Scope” Scenarios	Ensure the model does not hallucinate technical procedures when the situation is fundamentally non-technical.
Log Retrieval, Temporal Filtering, and Performance Stress	Test precision retrieval, filtering by time, and response performance under potentially large data requests.

Model performance was evaluated by four independent physicists using the same 10 standardized questions and a predefined scoring rubric. Reviewers were provided with the expected answers for each question to compare against model responses. These reference answers were established through manual review of the historical machine logs.

## Results

### Data collection

The custom Python web scraper extracted 1394 issue logs from five TrueBeam systems. Mean extraction time was approximately 15 min per machine, corresponding to about 3 s per issue. The final dataset comprised 5.44 MB of text.

### Data formatting & model indexing

**Fig. 2:**
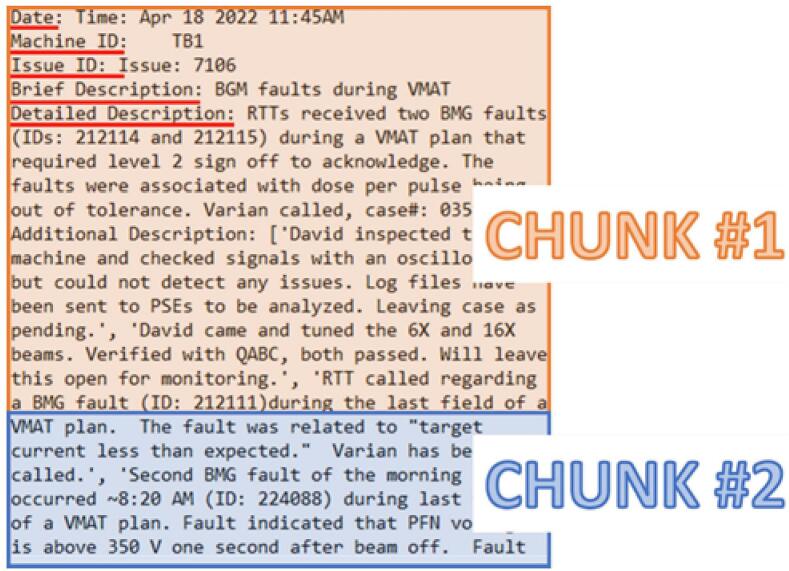
Each machine log was saved as a structured text file, which the model indexed into separate 1000-character chunks.

Following extraction and removal of non-UTF-8 characters, multiple file organizations were evaluated. Optimal performance was achieved when each issue was stored as an independent structured text file (Fig. 2). Information from separate files was never merged, allowing issues to be retrieved independently during response generation. Files shorter than 1000 characters were indexed as a single chunk, whereas longer files were divided into multiple chunks of 1000 characters or fewer, which were subsequently indexed into the model. Breaking individual files into multiple chunks did not degrade performance, provided the chunks-per-response parameter was sufficiently large to retrieve the complete log.

With this structure, indexing required 16.11 ± 0.52 min. Indexing time showed no measurable dependence on temperature or the number of chunks used per query (Pearson *r* < 0.3).

### Model parameter selection & testing

**Fig. 3:**
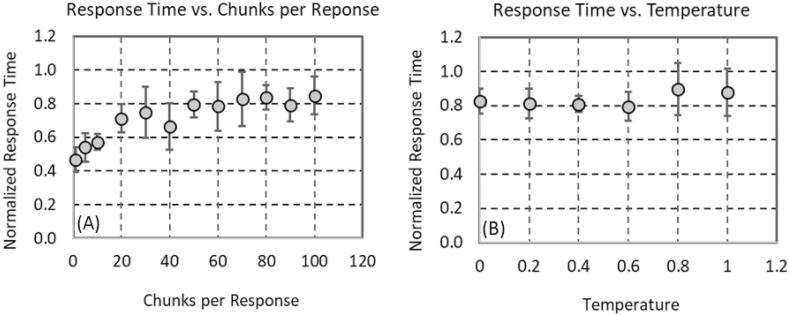
Impact of model parameters on response time: (A) varying chunks at temperature 0.4, (B) varying temperature with 40 chunks per response; results averaged over 10 questions.

The impact of model parameters on response time is shown in Fig. 3. The mean response time across all configurations and questions was 7.50 s +/− 2.50 s, with a maximum of 14.44 s.

**Fig. 4:**
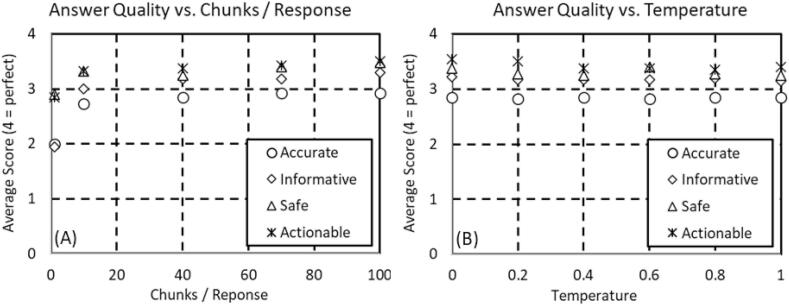
Effect of model parameters on response quality. (A) varying chunks at temperature 0.4, (B) varying temperature with 40 chunks per response; results averaged across scores from four physicists.

Temperature was set to 0.4, which provided the best balance between accurate interpretation of user queries and retrieval of relevant historical issues while limiting unrelated references. The number of chunks per response was fixed at 40. The final system prompt directed the model to determine whether a fault had occurred previously, reference prior events using identifiers such as dates, machine types, and issue numbers, and summarize past resolutions in a stepwise manner. The effect of parameter selection on response quality, as scored by physicists, is shown in Fig. 4. Overall, temperature and chunks per response had minimal effect on scored response quality, except when configured with a single chunk, which performed substantially worse than all other configurations.

**Fig. 5:**
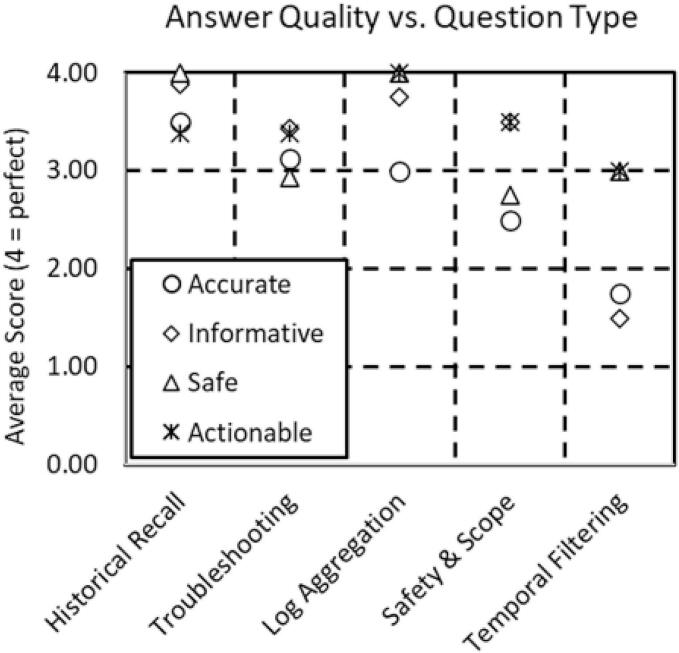
Impact of question type on response quality. Model temperature was 0.4, chunks per response 40; results averaged across four physicist scores.

Across models, performance was strong for recalling historical events spanning multiple logs and for recognizing queries outside the available data (Fig. 5). Responses were generally accurate and actionable; however, lower safety scores were observed, primarily due to ambiguity regarding the responsible party (field service engineers, physicists, or facilities staff) for specific actions. The models also showed limited ability to temporally filter logs and could not consistently retrieve information constrained to specified date ranges and failing to reliably identify the most recent logs from a given machine.

## Discussion

This study developed and evaluated a RAG-based troubleshooting chatbot was developed and evaluated using historical TrueBeam logs. Challenges arising during data collection, formatting, model indexing, and parameter optimization were identified and addressed; these are detailed in the following sections.

### Data collection

The first challenge was extracting data from a legacy database, where automated retrieval pathways were unclear due to the age of the system and staff turnover. While vendor-supported solutions can address such challenges, many institutions continue to rely on in-house tracking systems. From a data architecture perspective, commercial systems should facilitate rapid and efficient data acquisition, particularly for near real-time troubleshooting workflows where logs are updated regularly and models are re-indexed with current information.

Although timely updates support active fault resolution, data accuracy is critical. Incomplete or incorrect records can lead to irrelevant solutions, unnecessary downtime, or equipment damage. For example, dropdown menus used to identify subcomponents may become outdated, forcing users to leave fields blank or select incorrect options. Inclusion of “Other” and “Unknown” categories can mitigate this issue.

Compared with generic “Other” or “Unknown” categories, free-text entries overcome the limitations of outdated dropdown menus and provide users with greater flexibility to enter troubleshooting logs in a natural language format. However, free-text entries present additional challenges, as slight variations in terminology (e.g., “linac” vs. “LINAC”) can complicate the identification and grouping of similar troubleshooting logs. A key advantage of modern large language models (LLMs) incorporated into RAG systems is their ability to interpret variations in spelling and syntax, thereby grouping related terms appropriately. [19] Nevertheless, caution is warranted when applying this approach to equipment-specific data, as vendor- or model-specific terminology may be underrepresented in general LLM training data, potentially limiting accurate term association.

While free-text entries provide flexibility in data entry, this flexibility may also result in partially completed troubleshooting logs with missing information. One potential solution is a complementary documentation chatbot that guides users through a structured dialogue, automatically records the issue, and generates vendor notifications, while retaining the option for direct vendor contact if needed.

### Data formatting & model indexing

Although data retrieval methods are system-specific, this work highlights the critical role of post-retrieval formatting for successful RAG model indexing. Key factors included removing non-UTF-8 characters and storing each machine log in a separate structured file, which improved retrieval efficiency and reduced hallucinations. In this context, hallucinations refer to instances in which the model generates incorrect or unrelated information not grounded in the retrieved data, such as suggesting troubleshooting steps from unrelated historical issues when multiple logs are combined into a single chunk.

Indexing time was short enough to allow periodic re-indexing (e.g., nightly), though it will increase as more logs or supplementary resources, such as vendor manuals, in-house QA procedures, or national recommendations are incorporated. These considerations motivate the use of modular RAG architectures capable of handling both frequently updated data and static resources.

The concept of deploying multiple modular RAG systems within a unified AI framework has been explored previously, in which a single AI agent routes user queries to the most relevant RAG. [20] Within this paradigm, additional RAGs could be developed to house complementary machine resources such as vendor documentation and QA procedures. This approach offers two main advantages: physicists interact through a single interface for both documentation and troubleshooting, and only the relevant RAG needs re-indexing when new data are added. It also enables efficient maintenance, with a smaller RAG retrained frequently on recent issues and a larger archival RAG updated less often.

### Model strengths and limitations

Across models, recurring limitations were difficulties in maintaining correct procedural order and avoiding redundant steps. Many responses listed troubleshooting actions multiple times under slightly different names and occasionally proposed technically incorrect sequences, such as performing CBCT air normalization before blade calibration or IsoCal. While subtle, such errors can lead to invalid calibrations or wasted downtime, highlighting the need for explicit procedural hierarchy in RAG-based troubleshooting tools.

Some responses included hallucinated acronyms, misdefined terms, or outdated procedures. Others extrapolated from narrow scenarios, producing recommendations that were not broadly generalizable. These issues reflect a known limitation of language models: when documentation is sparse or ambiguous, the system may “fill in gaps” with plausible but incorrect information. Even minor inaccuracies can reduce user trust and increase cognitive burden in a clinical engineering context. This limitation may be further amplified when expanding the model to other clinical sites, as missing or non-localized information becomes more problematic in the presence of site-specific log structures, terminology, and procedures.

Safety concerns were also observed. Several sequences instructed users to enter service mode or deliver beam without explicitly ensuring patient removal, or advised manipulating internal components without escalation to field service engineers (FSEs) or facilities staff. Such recommendations pose personnel and equipment risks, emphasizing the need for clear role boundaries and escalation rules. Incorporating explicit safety steps in the model prompt and conducting a Failure Mode and Effects Analysis (FMEA) prior to deployment are recommended. [21] Such issues can be mitigated by amending the model prompt to explicitly include common-sense safety steps in responses.

Another limitation was poor differentiation between QA, calibration, and repair activities, as well as ambiguity regarding responsibility. Models occasionally conflated QA verification with calibration or included detector QA tasks in post-repair checks. Escalation pathways were inconsistent, sometimes suggesting physicist-led repairs that should be reserved for FSEs. While models performed well describing high-level workflows, they struggled when responsibility boundaries were critical.

Although many responses were technically correct, they were often verbose, repetitive, or included tangential information that detracted from clarity. Examples included pulling multiple nearly-identical logs from a single machine while ignoring others, referencing irrelevant historical or humorous entries, or appending unnecessary summaries and advice sections. In several cases, the models appeared to favor recall of nearby or familiar logs rather than comprehensive or representative retrieval. This behavior suggests that retrieval tuning and stricter relevance filtering are necessary to prevent distraction and ensure concise, actionable guidance.

These findings align with prior work on human-centered AI in oncology, which emphasizes that LLM-based clinical tools require transparency, bias mitigation, privacy protection, and continuous expert oversight. [22] Although this study focused on equipment troubleshooting rather than patient-facing oncology support, inaccurate recommendations could still affect patient care indirectly through treatment delays, staff safety risks, or inappropriate escalation pathways. Chow and Li also noted that LLM outputs may be shaped by biased or nonrepresentative datasets, a concern that parallels the influence of site-specific terminology and incomplete historical logs observed in this work. Accordingly, future troubleshooting systems should be deployed as clinical support tools with clear limitations, representative local data, feedback mechanisms, and ongoing safety monitoring.

While some of these error modes may be unique to LLMs, they parallel similar issues observed with other AI tools in radiation oncology (e.g., auto-contouring). As with any AI application in radiation oncology, appropriate education and training are essential, with emphasis that AI serves as a support tool and that the clinical practitioner remains ultimately responsible for the accuracy and safety of any AI output used in a manner that impacts patient care.

Despite these limitations, there were notable strengths. Models generally performed well when faults were highly variable and benefited from generalized troubleshooting approaches. Several responses demonstrated good distinction between transient and persistent faults, appropriate escalation to service, and clear summaries when the underlying documentation was strong. In many cases, responses were concise, accurate, and well-structured, indicating that with improved guardrails and domain constraints, RAG-based troubleshooting assistants can provide meaningful clinical value.

Furthermore, the rapid development of newer LLMs means that some of the limitations observed in this model may be mitigated in future versions. At the initiation of this study, GPT 4.1 was the most recent model available, whereas at the time of manuscript submission the latest version was GPT 5.5. Although newer models often address shortcomings of their predecessors, careful validation remains essential prior to clinical deployment. At minimum, they should undergo the same testing as earlier versions, with additional evaluation informed by failure modes identified in prior models during clinical use.

While the benefits and limitations of future models are difficult to predict, appropriate safety guardrails will be necessary to ensure safe clinical implementation. Integration with complementary clinical safety systems (e.g., checklists and decision-support tools) may further improve reliability, and these additional tools could be tailored to safeguard against some of the drawbacks of the chatbot, creating a hybrid system to maximize clinical utility.

Taken together, these findings suggest that while RAG-based troubleshooting tools show promise for accelerating fault resolution and reducing downtime, they require careful design to be safely deployed in clinical environments. Explicit encoding of procedural order, role-based permissions, safety-critical steps, and escalation boundaries is essential. Without these safeguards, even well-intentioned recommendations may introduce new risks. Future work should focus on structured troubleshooting frameworks, tighter coupling between logs and procedures, and systematic evaluation of safety omissions rather than purely technical correctness, while also assessing the generalizability of these findings across different machine log systems, vendor platforms, hospital sizes, and clinical staff demographics.

## Conclusion

This work demonstrates the feasibility of using a retrieval-augmented generation (RAG) architecture to support linear accelerator troubleshooting with institution-specific historical machine logs. By grounding responses in verifiable prior events, the GPT-4.1–based troubleshooting chatbot could rapidly summarize relevant institutional experience and provide context-aware guidance within clinically practical response times. Early deployment suggests such tools may enhance access to institutional memory, reduce cognitive load during high-pressure fault scenarios, and promote more consistent troubleshooting workflows across clinical staff.

At the same time, this study highlights critical limitations that must be addressed before broader clinical adoption. Issues with procedural sequencing, safety omissions, role ambiguity, and occasional hallucinated or outdated guidance emphasize the need for explicit safety guardrails, structured procedural hierarchies, and clear escalation rules. RAG-based tools should serve as decision-support aids rather than authoritative sources, complementing existing fault-management frameworks, such as AAPM Task Group 314, rather than replacing them. With careful design, ongoing validation, and formal risk analysis, AI-driven troubleshooting assistants may ultimately support safer, more efficient clinical operations and improve linac uptime.

## CRediT authorship contribution statement

**Cory Knill:** Writing – review & editing, Writing – original draft, Visualization, Validation, Supervision, Software, Resources, Project administration, Methodology, Investigation, Formal analysis, Data curation, Conceptualization. **Sean Devan:** Writing – review & editing, Validation, Methodology, Formal analysis, Conceptualization. **Charles Matrosic:** Writing – review & editing, Validation, Methodology, Formal analysis, Conceptualization. **Jill Moreau:** Writing – review & editing, Validation, Methodology, Formal analysis, Conceptualization. **Zheng Zhang:** Writing – review & editing, Validation, Methodology, Formal analysis, Conceptualization.

## Declaration of generative AI and AI-assisted technologies in the writing process

During the preparation of this work, the authors used ChatGPT in order to assist with copy editing of the manuscript. After using this tool/service, the authors reviewed and edited the content as needed and take full responsibility for the content of the publication.

## Funding

None

## Declaration of competing interest

The authors declare that they have no known competing financial interests or personal relationships that could have appeared to influence the work reported in this paper.
